# Improving Influenza Vaccination Coverage in Patients with Cancer: A Position Paper from a Multidisciplinary Expert Group

**DOI:** 10.3390/vaccines12040420

**Published:** 2024-04-16

**Authors:** Paolo Bonanni, Michele Maio, Giordano D. Beretta, Giancarlo Icardi, Alessandro Rossi, Saverio Cinieri

**Affiliations:** 1 Department of Health Sciences, University of Florence, Viale G.B. Morgagni 48, 50134 Florence, Italy; paolo.bonanni@unifi.it; 2 Medical Oncology, Department of Molecular and Developmental Medicine, University of Siena, 53100 Siena, Italy; 3 Department of Oncology, Center for Immuno-Oncology, Azienda Ospedaliero Universitaria Senese, 53100 Siena, Italy; 4 Medical Oncology Unit Pescara Hospital, Via Fonte Romana 8, 65124 Pescara, Italy; giordano.beretta@asl.pe.it; 5 Department of Health Sciences, University of Genoa, Via Pastore 1, 16132 Genoa, Italy; icardi@unige.it; 6 Hygiene Unit, Ospedale Policlinico San Martino IRCCS Genoa, Largo Benzi 10, 16132 Genoa, Italy; 7 Giunta Esecutiva SIMG, Via del Sansovino 172, 50142 Florence, Italy; rossi.alessandro@simg.it; 8 Medical Oncology and Breast Unit, Ospedale Perrino, S.S. 7 per Mesagne, 72100 Brindisi, Italy; saverio.cinieri@asl.brindisi.it

**Keywords:** cancer patient, COVID-19, immunosuppressed patient, influenza, vaccine

## Abstract

Patients with cancer can be immunocompromised because of their disease and/or due to anticancer therapy. In this population, severe influenza virus infections are associated with an elevated risk of morbidity and mortality. Influenza vaccination is therefore highly recommended in cancer patients, including those receiving anticancer therapy. However, vaccination coverage remains far below the recommended target for vulnerable subjects. Six specialists in oncology, hematology, immunology, and public health/vaccinology convened with the objective of developing strategies, based on evidence and clinical experience, for improving influenza vaccination coverage in cancer patients. This viewpoint provides an overview of current influenza vaccination recommendations in cancer patients, discusses barriers to vaccination coverage, and presents strategies for overcoming said barriers. New immunization issues raised by the COVID-19 pandemic are also addressed. Future directions include improving public education on influenza vaccination, providing the media with accurate information, improving knowledge among healthcare professionals, improving access to vaccines for cancer patients, co-administration of the influenza and COVID-19 vaccines, increased collaboration between oncologists and other health professionals, increased accessibility of digital vaccination registries to specialists, shared information platforms, and promoting immunization campaigns by healthcare systems with the support of scientific societies.

## 1. Introduction

Vaccines are the most powerful weapons against infectious diseases. Vaccinations are not only important for protecting individuals from diseases and their complications but also for limiting the circulation and transmission of pathogens [1]. Over the past two decades, vaccination programs have become increasingly complex due to the growing number of available vaccines, the need to establish the cost-effectiveness of recommended vaccinations, and uncertainties regarding vaccine effectiveness and adverse effects. Additionally, the role of vaccinations has expanded from directly fighting acute and often deadly diseases to the prevention of infection-related complications (e.g., human papillomavirus (HPV), where vaccination is recommended for the prevention of cervical cancer [2]), which is more difficult to quantify and appreciate [1]. Furthermore, due to the 2020 pandemic of coronavirus disease 2019 (COVID-19) and the subsequent rapid development of vaccines against severe acute respiratory syndrome coronavirus 2 (SARS-CoV-2) [3], vaccination programs and immunization schedules will require extensive revision.

Regarding immunization against seasonal influenza, safe and effective influenza vaccines have been available for over 60 years at the time of this article. According to the World Health Organization (WHO), vaccination is the most effective strategy for the prevention of seasonal influenza and its complications [4]. The morbidity and mortality burden of influenza-related complications on high-risk individuals, including those with impaired immune function, can be substantial and is well documented [5].

Vaccination against influenza in patients with cancer is a serious issue that presents many challenges, mainly in association with the immunocompromised state of these patients [6]. Owing to disease and/or treatment-associated immunosuppression, patients with cancer are vulnerable to infectious diseases and are at high risk of developing infection-related complications, including those associated with influenza [7]. Vaccination against common preventable diseases, including seasonal influenza, is therefore highly recommended in these patients [5,8,9,10,11,12]. However, evidence suggests that the rates of influenza vaccination in this high-risk group are far below the recommended goal of ≥75% coverage [13,14]. There are many reasons for these suboptimal vaccination rates, including cultural and practical reasons. For example, negative sentiments about vaccination on social media, as well as the widespread attitude that influenza is neither serious nor as harmful as other infectious diseases, are associated with lower vaccination rates [15]. Moreover, some people have expressed reluctance to receive the influenza vaccine because of concerns about its safety or efficacy [16], access to healthcare, and the cost of vaccination. In addition, the highly polarized stance of anti-vaccine conversations among social media users contributes to the issue of vaccine hesitancy [16]. In patients with cancer, the ability to generate an immune response to influenza vaccination largely depends on the time of vaccine administration in relation to chemotherapy treatment schedules and in relation to other antitumor treatments, as well as the type of vaccine (i.e., safety considerations dictate that only inactivated vaccines are indicated for patients with solid tumors receiving myelosuppressive chemotherapy) [6]. Thus, several important aspects need to be evaluated with respect to the clinical benefits of influenza vaccination during the management of patients undergoing systemic chemotherapy for cancer. Ideally, this evaluation would be a multidisciplinary decision-making process that involves all specialists caring for these patients.

## 2. Materials and Methods

Between January and May 2021, a group of six specialists from the fields of oncology, hematology, immunology, and public health/vaccinology convened in two web-based meetings to discuss issues related to influenza vaccination in patients with cancer in Italy, and the strategy based on evidence and clinical experience to improve vaccination coverage and its implementation. Participants were selected as experts in the aforementioned fields and acted as representatives of scientific societies. Each meeting was 3 h in duration and based on a predetermined agenda (Table S1). During each meeting, best practices were shared, and all participants were involved in discussions regarding the topics presented.

This paper reports the results of these two meetings and provides an overview of the specific issues discussed, including current influenza vaccination recommendations in Italy (Section 3 and Section 4), the barriers against ideal vaccination coverage in patients with cancer (Section 5), and practical suggestions on how to improve vaccination coverage based on these identified barriers (Section 6). New immunization issues raised by the COVID-19 pandemic and how the influenza and COVID-19 vaccination campaigns may help each other are also addressed (Section 7).

## 3. Annual Vaccination Plans and Influenza Vaccines

Based on WHO recommendations, national health systems publish an annual vaccination calendar that reports all recommended vaccinations. Such calendars are continuously updated to include novel vaccines and to reflect changes in the epidemiology of infectious diseases, although the Ministry of Health in Italy is slower than other national health systems in updating the National Vaccination Calendar, partly due to the fact that, historically, the calendar was strictly connected to the Triennial National Vaccination Plan and followed its updates [17]. Since 2022, the vaccination calendar has been independent of the new Plan, hopefully allowing the Ministry of Health to include important new vaccines without delays. The main objective of national vaccination plans is to increase the number of people being vaccinated, rather than increasing the number of vaccines included in annual calendars [18,19]. Thus, great effort is devoted to identifying individuals at risk of developing infection-related complications. Recommended vaccination schedules for high-risk individuals are usually developed in collaboration with specialists from relevant medical areas; general practitioners and pediatricians play a pivotal role in the promotion of vaccinations.

The WHO Strategic Advisory Group of Experts (SAGE) updates influenza vaccination recommendations annually and identifies individuals at risk of influenza-related complications. These typically include pregnant women and women up to 2 weeks postpartum, children under the age of 59 months, individuals aged less than 19 years on long-term aspirin- or salicylate-containing medications, individuals with a body mass index of ≥40 mg/kg^2^, older adults (≥65 years), and individuals with underlying health conditions such as chronic cardiac disease, asthma, chronic pulmonary disease, chronic renal disease, liver disease, hematological diseases, metabolic disorders, endocrine disorders (e.g., diabetes), neurological and neurodevelopmental disorders, and immunosuppressive disorders (e.g., HIV/AIDS) [4,20]. Influenza vaccination is also highly recommended for individuals at increased risk of exposure to or transmission of influenza virus, which includes healthcare workers and family members/caregivers of vulnerable individuals [4]. Due to the high mutation rate of the influenza virus, vaccine composition is also updated annually by the WHO based on virus strains in circulation in the prior winter season in the opposite hemisphere [21]. In the European Union/European Economic Area, the recommended rate of influenza vaccination coverage is ≥75% in high-risk groups [22].

The recommendations on influenza vaccination for the 2021–2022 season, issued by the WHO Regional Office for Europe, included the co-administration of influenza and COVID-19 vaccines, based on initial evidence supporting its feasibility and safety [4]. Furthermore, co-administration is highly recommended to combat the increasing burden these concomitant infections have on patients and healthcare systems [4].

## 4. Influenza Vaccination for Patients with Cancer

### 4.1. Rationale and Evidence

Influenza viruses are transmitted efficiently via small airborne aerosols or larger respiratory droplets, by direct person-to-person contact, or via contaminated surfaces and are responsible for a large global healthcare burden that accounts for millions of lost school or work hours, as well as millions of physician visits each year [23]. Influenza viruses can be particularly severe for certain populations such as young children, elderly people, immunocompromised individuals, and those with underlying diseases, with sometimes fatal consequences [23]. In particular, the consequences of influenza-related infections are dire for people with cancer, especially those undergoing chemotherapy [17]. According to the WHO, seasonal influenza is responsible for an estimated 650,000 deaths globally each year on average [24], although some researchers believe that global influenza-associated mortality estimates may underestimate the disease burden [25].

Patients with solid tumors and hematologic malignancies can be immunocompromised because of their disease and/or as a consequence of anticancer therapy [26]. It is well recognized that the efficacy of anticancer agents involves direct cytostatic/cytotoxic effects and that chemotherapeutic agents can promote the (re)activation of tumor-targeting immune responses by increasing the immunogenicity of malignant cells or by inactivating immunosuppressive circuits established by progressing neoplasms [27]. Conversely, these cytostatic/cytotoxic effects of chemotherapeutics also induce immunosuppressive side effects that are commonly associated with bone marrow suppression, including lymphopenia and thrombocytopenia, and other hematologic issues such as neutropenia [28], while radiation-induced lymphopenia is a common side effect of radiotherapy, with some patients experiencing profound and enduring depletion of circulating lymphocytes after irradiation [29].

The immunosuppressive activity of cytotoxic agents, targeted therapies, and systemic corticosteroids in patients with cancer suppresses their innate immune responses and increases their risk of infections from various pathogens, including respiratory infections that typically manifest as a common cold in immunocompetent people but can be life-threatening for those who are immunocompromised [30,31]. In particular, chemotherapy- and radiation-induced neutropenia is a well-recognized risk factor for cancer-associated bacterial pneumonias and secondary complications and infection-associated deaths in cancer patients compared with the general population [32].

The literature has documented the fact that cancer patients infected with influenza are more likely to experience serious complications than cancer patients without influenza [31], and severe influenza virus infections requiring hospitalization are associated with increased morbidity and mortality in cancer patients compared with the general population [7]. It is of great concern that patients with cancer are highly susceptible to developing pulmonary complications of influenza, and particularly the development of bacterial pneumonias, which are associated with disproportionate morbidity and mortality in this population [32,33].

Notably, fatality rates ranging from 5% to 33% have been reported for cancer and hemopoietic stem cell transplant (HSCT) recipients with influenza [31,34,35,36]. Furthermore, influenza infection often delays the initiation of anticancer treatment or interrupts ongoing treatment, worsening the oncologic outcome [6].

In patients with cancer, the degree of immunosuppression can vary from mild to severe, an aspect that should be considered when deciding whether or not to vaccinate against influenza [37]. For instance, evidence suggests that this immunosuppressive state impacts the development of post-vaccination antibodies, with cancer patients not generating the same antibody responses as immunocompetent individuals [38]. Reduced antibody production against common pathogens has been well documented in patients with multiple myeloma [39]. Notably, reports have documented much higher serious infection rates in patients with multiple myeloma compared with those with either Waldenstrom’s macroglobulinemia or monoclonal gammopathy of undetermined significance (MGUS) [39]. In one study, patients with multiple myeloma or Waldenstrom’s macroglobulinemia had significantly lower baseline antibody titers to pneumococci compared with healthy controls, which reflects their susceptibility to pneumococcal infections and emphasizes the significance of vaccination for these patient populations [39].

Live vaccines are not recommended for immunocompromised patients [37]; inactivated or subunit vaccines are generally preferred. However, depending on the degree of immune dysfunction, inactivated vaccines may not induce an adequate immune response in patients with cancer. Before initiating immunosuppressive treatments or performing transplantation procedures, the patient’s immunization status with regard to required vaccines should be part of routine assessments [37]. According to the guidelines issued by the Infectious Diseases Society of America (IDSA) in 2013, recommended vaccinations should be planned prior to immunosuppressive treatments [40]. With regard to influenza, the limited recommended time for vaccine administration (October–December in the Northern Hemisphere) may overlap with unfavorable immunity conditions that could undermine immunization. In such cases, alternative strategies can be adopted, as described in detail in the 2013 IDSA guidelines [40].

Overall, the published evidence suggests that influenza vaccines are safe and, with some adjustments (e.g., increased dose), are capable of inducing an effective immune response and reducing the impact of influenza in cancer patients treated with a wide range of anticancer therapies, such as cytotoxic chemotherapy, targeted therapy, and immunotherapy [41,42,43,44,45,46,47,48,49,50]. The recent Italian multicenter INVIDIa-2 study prospectively evaluated 1188 patients with advanced cancer treated with immune checkpoint inhibitor immunotherapy; 581 of these patients (48.9%) had received influenza vaccination [45]. The incidence of influenza-like illness (primary endpoint) was similar in vaccinated and unvaccinated patients. However, vaccinated patients had significantly fewer influenza-related complications (11.8% vs. 38.3% in unvaccinated patients) and a consequent need for influenza-like illness-related intravenous treatments (11.8% vs. 29.8%, respectively). Additional treatments targeting influenza complications may further delay surgery or other pharmacological interventions in patients with cancer. A clear illustration of this point to patients is likely to increase their motivation to comply with vaccination procedures.

### 4.2. Current Recommendations

Specific evidence-based recommendations on influenza vaccination in patients with cancer were issued in 2014 by the Italian Society of Medical Oncology (AIOM) [8]. These recommendations, which are regularly updated [9,10,12], state that influenza vaccination is an effective, safe, non-invasive, and relatively inexpensive measure to prevent influenza-associated complications in patients with cancer [8]. According to the AIOM guidelines, influenza vaccination should be given to all patients with cancer, regardless of whether they are receiving anticancer therapy or not, including those receiving biologics for solid tumors. While both trivalent and quadrivalent vaccines can be administered, use of the latter appears to be increasing. The introduction of personalized, or at least targeted, vaccination programs to groups of patients with similar characteristics and the type of influenza vaccines could elevate vaccination effectiveness in patients with cancer. The ideal timing for vaccination in relation to cancer therapy is currently unknown. Family members and caregivers of patients with cancer should also be vaccinated against influenza to efficiently block virus spread.

The COVID-19 pandemic further highlights the importance of influenza vaccination in patients with cancer, as the two infections have similar initial symptoms, and the occurrence of symptoms that suggest a SARS-CoV-2 infection may preclude these patients from receiving appropriate care and anticancer treatment. Furthermore, concomitant respiratory infections may have synergistic effects, although the interactions between SARS-CoV-2 and other respiratory viruses and their effects on disease severity and new viral variants are not yet fully understood [51].

Despite growing evidence supporting the effectiveness of influenza vaccination in cancer patients [41,43,44,45,46,47,48,49,50] and current recommendations, the rate of vaccination in cancer patients remains far below the recommended coverage rate of ≥75% in many countries [52]. For example, in the retrospective INVIDIa-2 study mentioned above, fewer than 50% of the cancer patients enrolled had received an influenza vaccination [45]. This low uptake may reflect, in part, a general trend for suboptimal vaccination rates in the wider population, as shown by recent national influenza vaccination survey findings from 49 WHO European Member States covering the period 2008/2009 to 2014/2015 showing suboptimal influenza vaccination uptake across the European Region, despite widespread national policy recommendations [52]. A potential method to increase vaccine coverage may be to expand vaccination recommendations towards individuals living and/or working with cancer patients. Increased vaccination rates in individuals around cancer patients may increase patient motivation for vaccination as well.

### 4.3. COVID-19 Vaccination in Patients with Cancer

SARS-CoV-2/COVID-19 is associated with increased morbidity and mortality in the cancer population [53,54,55,56]. Data on the efficacy and safety of COVID-19 vaccines in this population, which is generally excluded from trials evaluating COVID-19 vaccines, are limited but steadily growing [38,57,58,59]. Emerging findings are promising, warranting further research. Several practical issues, including the optimal timing of vaccination with respect to anticancer therapies, remain to be established. These uncertainties have been addressed in guidance issued by the COVID19 and Cancer Clinical Trials Working Group, which advises that patients with cancer, including those enrolling into or currently enrolled in oncology clinical trials, should be prioritized for COVID-19 vaccination [60]. The guidance recommends that for such individuals, the vaccination schedules can vary according to the type of trial, disease, and cancer treatment [60]. The only exception to this scheduling applies to patients on clinical trials involving transplantation or adoptive cell treatments; in such cases, vaccine administration may need to be delayed for 3 months or more to enable these patients to regain adequate immune function [60]. Many scientific societies have recognized the importance of vaccinating all patients with cancer, including those receiving active therapy, against COVID-19 [60]. These scientific societies include the American Society of Clinical Oncology (ASCO), the Association of American Cancer Institutes (AACI), the American Association for Cancer Research (AACR), the AIOM, the European Society for Medical Oncology (ESMO), the Society for Immunotherapy of Cancer (SITC), and the Spanish Medical Oncology Society (SEOM) [60].

Approval of the COVID-19 vaccine booster dose in 2021 and the inclusion of cancer patients among the priority groups raised questions about how the booster program may be implemented without disruption or delay to the influenza vaccination program. In the 2020–2021 season, the Advisory Committee on Immunization Practices (ACIP) of the US Centers for Disease Control and Prevention (CDC) recommended an interval of at least 2 weeks between COVID-19 and any other vaccinations [61]. This measure of caution is no longer deemed necessary, and the CDC, along with the European Centre for Disease Prevention and Control (ECDC), the UK Joint Committee on Vaccination and Immunization (JCVI), and other public health authorities have stated that COVID-19 and influenza vaccines can be co-administered [62,63]. According to the WHO-Europe recommendations on influenza vaccination for the 2021–2022 season, “the co-administration of COVID-19 vaccines with certain seasonal influenza vaccines is acceptable in terms of immunogenicity and reactogenicity”, based on the emerging evidence [4,64,65]. The WHO guidelines have also underlined the convenience of co-administration in terms of reduced number of visits, timely protection against both infections, and the possibility of successfully implementing both vaccination programs [4].

## 5. Barriers to Influenza Vaccination

Many barriers to influenza vaccination have been identified. Primarily, the need for influenza vaccination is not widely recognized. This lack of recognition is illustrated by a Polish survey that explored the influence of the COVID-19 pandemic on the decision to be vaccinated against seasonal influenza in a cohort of 236 cancer patients who were receiving chemotherapy between June and September 2020 [66]. In that survey, half (50.8%) of the respondents were unsure about the effectiveness of the influenza vaccine; only 33.5% of the respondents believed that the influenza vaccine was effective, while only one in five respondents (19.9%) thought it was safe [66]. Although general practitioners have played a major role in improving rates of vaccinated cancer patients, a general lack of medical education around vaccination still constitutes a major obstacle to the widespread administration of influenza vaccines. It is possible that the COVID-19 pandemic and its associated vaccination campaigns may improve the public’s general attitude towards vaccines; however, ‘vaccine fatigue’ may remain a challenge to increasing the uptake of any vaccine, including that for influenza [67,68].

Despite influenza vaccines having been used successfully for decades, there is still a sense of uncertainty about their efficacy and safety. Physicians may have an important role in fostering this misperception, as they often fear side effects, disregard benefits, and fail to convey both a convincing and clear message about the elevated risk of morbidity and mortality to vulnerable patients and the favorable benefit-risk balance of vaccination. Indeed, healthcare workers’ own influenza immunization status may impact vaccination rates in patients with cancer. While a lack of correlation between vaccination status among healthcare workers and patient vaccination has been reported in a general population cohort in Spain [69], data in this regard that are specific to cancer patients would be useful.

Several practical issues may also constitute barriers. In many countries, including Italy, vaccinations are not integrated into the care pathway of patients with cancer, which significantly complicates access to vaccination. Problems with vaccine availability (e.g., as was experienced during the 2020–2021 influenza season in Lombardy, Italy) may also limit the number of cancer patients being vaccinated.

Finally, anti-vaccination movements also contribute to reluctance in accepting vaccinations in Italy and elsewhere. Although anti-vaccination groups are a minority (2% of the Italian population), their arguments are widespread and easily accessible online. Consequently, individuals who have never received clear and correct information from healthcare professionals and are therefore unsure about vaccination benefits may be easily influenced.

## 6. Strategies to Overcome Barriers

There is an urgent need to greatly improve and intensify the quantity and quality of available information and education surrounding the effectiveness and safety of vaccines, targeting both the public and healthcare professionals. Educational efforts should not aim to convince anti-vaccination groups (minorities) but should instead try to provide clearer and more comprehensive information to people who are simply uncertain about vaccination benefits and constitute a much larger proportion of the general population [70]. Information on the benefits of vaccination should be introduced early in the education system (primary school) and should have a more prominent place in science and in the medical curriculum within secondary and tertiary education [71,72].

Regarding influenza vaccination in patients with cancer, general practitioners need to be better informed about the oncologic setting, including main treatment options (chemotherapy, targeted therapy, immunotherapy), type of disease, and patient characteristics. A survey conducted during the period 2010–2011 examined the practice of general practitioners towards influenza vaccination in patients receiving ongoing chemotherapy for breast cancer or colorectal cancer in The Netherlands [73]. The survey findings revealed that 49 of the 107 (46%) participating general practitioners were apparently not aware of the national guidelines recommending annual influenza vaccination for all patients treated with chemotherapy or other immunosuppressive agents [73]. Moreover, approximately half (48%) of the general practitioners stated that they would not approach patients treated with chemotherapy, reasoning that vaccination against influenza is the responsibility of the treating medical oncologist [73].

Improving access to vaccination is also crucial. Patients with cancer often do not know where and when to receive recommended vaccinations. For instance, vaccines could be administered when patients visit hospitals for therapies or follow-up visits. What has been learned and achieved with the COVID-19 vaccination campaign will hopefully promote the development of strategies for widespread immunization against other infectious diseases. Influenza vaccination by appointment (widely implemented in the 2020–2021 season for safety reasons) may help underline the importance of vaccination to individual patients, improve organizational issues, and eventually lead to the implementation of influenza and COVID-19 vaccine co-administration.

An increasing number of clinics in Italy are implementing new strategies for improving immunization rates in high-risk patients. A good example of this is the San Martino Hospital in Genova. This hospital has facilitated increased access to vaccination by establishing a dedicated vaccination center. This center provides all recommended vaccines, including influenza vaccines, to patients belonging to high-risk groups (mostly patients receiving HSCT). Patients are vaccinated during their stay in hospital or at discharge. The introduction of this strategy has resulted in a substantial shortening of time from HSCT to administration of the first post-transplant vaccination. Multidisciplinary collaborations among many specialists have been crucial in achieving these results. Other factors contributing to increased vaccine coverage include the active offer of free vaccination to patients; the creation of digital vaccination registries; offers of in-hospital vaccinations, or at centers of diagnosis and treatment; sharing of immunization programs with other specialists; and specific inclusion of vaccinations in disease care programs. To improve multidisciplinary collaborations, the Public Health Department has developed a platform which may be used by all hospital units to provide vaccination consulting. Notably, the strategy developed by the San Martino Hospital has also been very useful in managing COVID-19 vaccination in vulnerable patients. It is hoped that the success of this approach will encourage the adoption of similar strategies by other hospitals and regions. However, it should be noted that regulatory and administrative differences between regions and even between local districts within the same region may necessitate vaccination strategies being custom-made for each hospital. Furthermore, it should be emphasized that such strategies require a multidisciplinary approach, which should include an educational phase and collaboration of all specialists involved in the care of patients with cancer (e.g., radiotherapists, surgeons, nurses).

Suggested measures for overcoming barriers to influenza vaccination in cancer patients are summarized in Table 1.

## 7. Future Directions

As mentioned above, improving public education on influenza vaccination and the risk of infection in vulnerable groups is crucial, as is providing the media with accurate information and improving knowledge among healthcare professionals through continuing medical education programs. Increased access to vaccines may be facilitated by ensuring that vaccinations are delivered within the care program of oncologic patients, for example during hospital stays, at discharge from hospital, or during scheduled follow-up visits. Given that COVID-19 vaccination is also highly recommended for cancer patients [74] and that COVID-19 vaccination campaigns have been effective, influenza vaccination could be administered with COVID-19 vaccination during the same visit [75,76,77]. This should simplify operational issues considerably and increase the number of cancer patients receiving the recommended vaccinations. Co-administration of influenza with pneumococcal vaccines (conjugate and polysaccharide) is supported by specific studies, as well as influenza together with zoster vaccines, in addition to COVID and influenza vaccines, as already mentioned [78]. However, in general, according to the US Pink Book [79], all co-administrations are possible, unless specifically discouraged due to safety or immunological interference issues. It is recommended that oncologists and all other specialists involved in the care of patients with cancer should network and collaborate more closely with general practitioners so that information surrounding vaccinations is consistent and reinforced. Digital vaccination registries that are accessible to all specialists involved in the care pathway of patients with cancer and shared information platforms facilitate easier multidisciplinary collaboration, help to identify candidates for vaccination, and aid in implementing vaccination programs, as shown by the COVID-19 pandemic. Finally, immunization campaigns should be promoted by healthcare systems with the support of scientific societies that can play a crucial role in promoting initiatives to specialists so that they can provide the best support to patients and overcome barriers to vaccination.

## Figures and Tables

**Table 1:** Suggestions on how to reduce the barriers to influenza vaccination in patients with cancer.

Barrier	Strategy
• Need for vaccination is not recognized; uncertainty about the efficacy and safety of influenza vaccines; anti-vaccination movements contribute to vaccine hesitancy	• Improve the information to the target population (cancer patients, their families, and caregivers) and to the media, with clear and comprehensive messages that explain the risks of influenza for vulnerable subjects and the favorable benefit-risk profile of influenza vaccination;
	• Vaccination campaigns should be promoted by healthcare systems with the support and engagement of scientific societies
• Lack of medical education around vaccination	• University curricula in medicine and continuing education programs for physicians should have courses devoted to vaccines and immunization.
• Vaccination is not integrated into care pathway	• The access of cancer patients to influenza vaccines can be facilitated by scheduling the vaccination within the oncologic care pathway, for example during planned follow-up visits;
	• Co-administration of vaccines is feasible and may simplify operational issues. The co-administration of influenza vaccine and COVID-19 vaccine, in particular, may increase the number of cancer patients being vaccinated against seasonal influenza;
	• The collaboration between oncologists and general practitioners needs to be increased, for example by creating networks, so that messages about vaccination are consistent and reinforced;
	• Digital tools (e.g., digital vaccination registries, shared information platforms) may be very powerful in promoting multidisciplinary collaborations and should also be used for identifying candidates for influenza vaccination.

## Data Availability

No underlying data were collected or produced in this study.

## Supplementary Materials

The following supporting information can be downloaded at: https://www.mdpi.com/article/10.3390/vaccines12040420/s1, Table S1: Agendas for two web-based advisory board meetings regarding strategies for improving influenza vaccination coverage in patients with cancer in Italy.
