# Supplementary material for: Improving Influenza Vaccination Coverage in Patients with Cancer: A Position Paper from a Multidisciplinary Expert Group
Source: Vaccines (Basel). 2024 Apr 16;12(4):420. doi: 10.3390/vaccines12040420 (PMC11053698; doi:10.3390/vaccines12040420)
Supplement: Supplementary file 1 [file vaccines-12-00420-s001.zip › vaccines-2862574-supplementary.pdf]

Table S1. Agendas for two web-based meetings regarding strategies for improving influenza vaccination coverage in patients with cancer in Italy.

Meeting	Time	Topic
Meeting 1	4:00 pm – 4:20 pm	Welcome and Introduction
	4:20 pm – 4:40 pm	Overview of the National Vaccination plan
	4:40 pm – 5:00 pm	National strategy for influenza vaccination
	5:00 pm – 5:40 pm	What to do for the cancer patient?<br>– AIOM recommendations<br>– Discussion
	5:40 pm – 6:10 pm	Cancer patient and current barriers: how to overcome them?<br>– Best practice sharing-network – examples?<br>– Special populations:<br>• Hematology or immunocompromised patients<br>• Immune oncology (monoclonal antibodies or cell therapies)<br>• COVID landscape
	6:10 pm – 6:50 pm	Proposals for a draft action plan
	6:50 pm – 7:00 pm	Conclusions and next steps
Meeting 2	4:00 pm – 4:10 pm	Welcome and Introduction
	4:10 pm – 4:30 pm	Best practices sharing-network – examples?
	4:30 pm – 5:30 pm	What actions to take to improve awareness of the importance of influenza vaccination among cancer patients (round table discussion)
	5:30 pm – 6:00 pm	Other areas of collaboration between the company and stakeholders: data generation<br>Overview of clinical data on recombinant influenza vaccine
	6:00 pm – 6:50 pm	Discussion
	6:50 pm – 7:00 pm	Concluding notes and next steps

AIOM, Associazione Italiana di Oncologia Medica (Italian Association of Medical Oncology).
